# Advances and challenges of prenatal interventions for fetal tachyarrhythmias

**DOI:** 10.3389/fped.2024.1509158

**Published:** 2024-12-17

**Authors:** Jiao Tang, Pujue Huang, Xue Deng, Lijuan Zhao, Yang Zhai, Tao Wang

**Affiliations:** ^1^ Department of Pediatrics, West China Second University Hospital, Sichuan University, Chengdu, China; ^2^ Key Laboratory of Birth Defects and Related Diseases of Women and Children, West China Second University Hospital, Sichuan University, Chengdu, China; ^3^ Department of Pediatrics, First People’s Hospital of Longquanyi District, Chengdu, China; ^4^ Department of Pediatrics, Western Theater General Hospital, Chengdu, China; ^5^ Department of Pediatrics, Chengdu Women and Children’s Central Hospital, Chengdu, China

**Keywords:** fetal arrhythmias, tachyarrhythmias, prenatal intervention, transplacental drug therapy, sotalol

## Abstract

It is estimated that 1%–2% of pregnancies are complicated by fetal arrhythmias, with most arrhythmias considered benign and not requiring further treatment or intervention. However, persistent tachyarrhythmias can lead to fetal heart failure, preterm birth, stillbirth, and increased risks during the perinatal period. Therefore, timely treatment during pregnancy is often necessary. Currently, prenatal treatment for fetal tachyarrhythmias (FTs) is primarily drug based, aiming to restore normal fetal heart rate, prevent or reverse fetal heart failure, and avoid adverse outcomes such as preterm birth and stillbirth. Despite decades of clinical experience, the lack of prospective, multicenter randomized clinical trials on the safety and efficacy of drugs means that there is still no universally accepted prenatal treatment regimen for FTs, and treatment relies on series of observational studies or clinical cases. Moreover, all drug treatments carry potential risks to the mother, fetus, and pregnancy, hence the need for more clinical diagnostic and therapeutic experience to provide more clinical evidence for prenatal treatment of FTs.

## 1 Introduction

The heart rhythm of a normal fetus is regular, with a rate of 110–160 beats per minute. Fetal tachyarrhythmias (FTs) is defined as a fetal heart rate persistently exceeding 180 beats per minute in the absence of uterine contractions. The most common types are supraventricular tachycardia (SVT) and atrial flutter (AFL), while ventricular tachycardia (VT) is rare (1). FTs are important causes of intrauterine fetal nonimmune edema, preterm birth, and increased perinatal morbidity and mortality. Compared to fetuses with intermittent SVT or AFL, those with persistent SVT or AFL (tachycardia >50% of the time or >12 h/day) or fetal edema have poorer outcomes, with increased mortality and risk during the perinatal period (2). A 10-year retrospective cohort study showed that compared to nonedematous fetuses, edematous fetuses have a higher rate of *in utero* treatment but significantly lower survival rates, earlier delivery, lower cardiovascular scores at diagnosis and birth, lower birth weight, and higher termination of pregnancy rates (3). The American Heart Association recommends that (4), in addition to near-term fetuses, *in utero* treatment is recommended for all fetuses with persistent tachyarrhythmias, with or without edema, or with intermittent tachyarrhythmias associated with heart failure or edema. A study from Japan showed that (5), compared to untreated fetuses, *in utero* treatment significantly reduces the rates of cesarean section, preterm birth, and neonatal arrhythmias. In ∼80% of cases, transplacental drug therapy is effective, allowing for a smooth postponement of delivery into the third trimester (6).

Currently, there is no consensus on the optimal treatment strategy for FTs. Prenatal treatment primarily consists of drug therapy, and digoxin, flecainide, sotalol, amiodarone, and other drugs are supported by clinical data as first-line treatments (7–10) (Table 1). However, the treatment of FTs still faces many unknown issues, such as drug selection, dosage, effective routes of administration, and monitoring of drug toxicity and side effects.

**Table 1:** Antiarrhythmic drugs.

	Digoxin	Flecainide	Sotalol	Amiodarone
Indications	short VA SVT without FH	short VA SVT without or with FH	AF without FH, long VA SVT	refractory SVA
Transplacental medication	LD: 500 mg q12 h for 2 d MD:375–750 *μ*g/d q8 h to q12h	200–400 mg/d q8h–q12h	160–480 mg/d q8h–q12h	LD: 1,200–2,400 mg/d q6 h for 2d MD: 200–400 mg/d
Direct fetal medication	Intramuscular 88 μg/kg fetal weight q12h repeat 2 times	Intraperitoneal Case report: 2 mg/kg fetal weight	Not applicable	Intraperitoneal 2.5–5.0 mg/kg fetal weight
Goal drug levels	1.0–2.5 ng/ml	0.2–1.0 *μ*g/ml	<2.5 mg/L	1.0–2.5 *μ*g/ml
Combination therapy	Monotherapy failed, or SVA with FH: digoxin + sotalol, or digoxin + flecainide			Amiodarone + digoxin or flecainide
Adverse effects	nausea/vomiting/fatigue blurred vision low birth weight	CNS symptoms visual disturbances obstetric cholestasis	nausea/vomiting/fatigue low birth weight neonatal hypoglycemia	thyroid dysfunction photosensitivity rash thrombocytopenia fetal goiter neurodevelopmental concerns

Short VA SVT, short VA interval supraventricular tachycardia; long VA SVT, long VA interval supraventricular tachycardia; FH, fetal hydrops; SVA, supraventricular arrhythmia; LD, loading dose; MD, maintenance dose; CNS, central nervous system.

## 2 Types and etiology of FTs

### 2.1 Sinus tachycardia

Sinus tachycardia (ST) refers to a fetal heart rate of 180–200 beats per minute with normal atrioventricular conduction. ST is usually associated with intrauterine distress and underlying maternal diseases, including hyperthyroidism, anemia, fever, use of *β*-receptor agonists or stimulants, fetal hypoxia, chorioamnionitis, and intrauterine cytomegalovirus infection (1). The prenatal treatment strategies for ST focus on correcting the underlying causes, with good prognosis in most cases.

### 2.2 SVT

SVT is the most common FTs, accounting for 60%–90% of cases (11). The incidence of fetal edema in SVT is as high as 30%–40% (6, 9), making it a significant cause of fetal morbidity and mortality. SVT often occurs between weeks 24 and 32 of gestation, with a fetal atrial rate of 220–300 beats per minute and 1:1 atrioventricular conduction. The electrophysiological mechanism is mainly accessory pathway conduction forming a re-entrant circuit. Based on the relationship between ventriculoatrial (VA) interval and atrioventricular(AV) interval, SVT can be further classified into short and long VA SVT. Some studies found a significant increase in the incidence of fetal SVT during the COVID-19 pandemic (12). Abdallah et al. reported two cases of fetal SVT after maternal COVID-19 vaccination (13), possibly related to severe fetal hypoxia caused by SARS CoV-2 infection of the placenta (14) and immune metastasis and immune response of the placenta.

### 2.3 AFL

Approximately 30% of FTs are AFL (2), which often occurs in late gestation with a fetal atrial rate >300 beats per minute and an atrioventricular conduction ratio of 2:1, 3:1, or even 4:1. AFL is associated with myocarditis, anti-Ro (SSA), anti-La (SSB) autoantibodies, and congenital heart disease, with ∼15% of fetuses with AFL developing fetal edema (1, 4).

### 2.4 VT

VT is rare in the fetal period, accounting for <2% of FTs (15). The ventricular rate is between 180 and 300 beats per minute, with a ventriculoatrial ratio <1. Its electrophysiological mechanism is ectopic ventricular foci caused by inflammation or abnormal myocardial oxygen supply. VT can occur in fetuses with atrioventricular block, cardiac tumors, myocarditis, ventricular aneurysm, and cardiomyopathy (2). When VT coexists with sinus bradycardia or second-degree block, the possibility of long QT syndrome (LQTS) needs to be considered (1).

## 3 Treatment of FTs

In recent years, with the rapid development of fetal medicine, the intrauterine treatment methods for fetal diseases have gradually increased. However, prenatal treatment for fetal arrhythmias still mainly relies on drug therapy, mainly including: transplacental drug therapy; administration via umbilical vein injection; administration via the fetal abdominal cavity; administration via the amniotic cavity; and administration via fetal intramuscular injection. Transplacental drug therapy is the preferred approach, while other methods are limited in clinical application due to their invasiveness, and are only considered when the placental transfer rate of severe fetal arrhythmias is low.

### 3.1 Transplacental drug transport

#### 3.1.1 Treatment for SVT and AFL

##### 3.1.1.1 Digoxin

Digoxin is the longest-used medication for treating FTs. It has a high protein-binding rate, but its ability to cross the edematous placenta is poor, requiring larger and more frequent doses to achieve therapeutic levels in the fetus (16). A multicenter prospective study from Japan showed that (7), for treating short VA SVT without fetal edema, the single-drug relief rate of digoxin was 46.7%, while the total relief rate of digoxin combined with sotalol or flecainide was 86.7%; for AFL without fetal edema, the single-drug relief rate of digoxin was 59.3%, and the total relief rate of digoxin combined with sotalol or flecainide was 92.6%. The efficacy of digoxin treatment for long VA SVT patients is poor (7, 17). In cases of FTs with accompanying edema, the relief rate of digoxin also decreases significantly to <20%, and the occurrence of intrauterine or neonatal death in edematous fetuses treated with digoxin alone reaches 43% (9, 18).

##### 3.1.1.2 Flecainide

Flecainide is a lipophilic compound with no significant protein binding, and it has good placental transfer (16). Several studies have suggested that flecainide may be more effective than sotalol or digoxin, and fetal intrauterine death is rare (8, 9, 19). It has been reported that the relief rate of flecainide monotherapy for fetal SVT can be as high as 88.2% (20). Two meta-analyses have shown that (8, 19) flecainide and sotalol are superior to digoxin in cases of FTs with or without fetal edema. Especially, when fetal edema is present, the effectiveness of flecainide compared to digoxin is more apparent. Additionally, in cases of long VA SVT, flecainide is also superior to digoxin (9), and placental administration of digoxin combined with flecainide is an effective treatment (21). Therefore, flecainide monotherapy and combination of digoxin and flecainide should be considered the optimal strategies for FTs (22).

##### 3.1.1.3 Sotalol

Sotalol is rapidly absorbed and crosses the placenta almost completely (16). Studies have shown that compared with digoxin or flecainide, the use of sotalol alone or in combination with digoxin is associated with a higher rate of prenatal AFL termination. Sotalol can be recommended as the first-choice for treating fetal AFL (23). Jaeggi et al. (24) conducted a multicenter nonrandomized study on fetuses with AFL and found that sotalol was a better choice for treating fetal AFL. For long VA SVT, sotalol has a higher rate of successful conversion compared to digoxin or flecainide (7).

##### 3.1.1.4 Amiodarone

Amiodarone, either alone or in combination with other drugs, is a safe and effective approach for refractory FTs, even in the presence of fetal edema or cardiac dysfunction (25). It is worth noting that amiodarone is associated with maternal and neonatal thyroid dysfunction, and caution is needed regarding the potential risk of fetal neurodevelopmental issues (16).

#### 3.1.2 Treatment for VT

Fetal VT is rare. It should be noted that, while treating fetal VT, it is necessary to exclude the possibility of LQTS through fetal magnetocardiography (fMGG) (26). If LQTS can be ruled out, sotalol, amiodarone, or flecainide may be considered (16). Studies have shown that intravenous administration of magnesium sulfate to the mother can be used as a first-line option for fetal VT (2, 15, 27), but its use should be limited to within 48 h. Vaksmann et al. (28) suggested that beta-blockers should be used as first-line therapy for isolated fetal VT (i.e., without tumors or cardiomyopathy), as LQTS may be a potential cause of arrhythmia.

### 3.2 Direct fetal medication

For FTs that fail to respond to placental drug transfer therapy, direct fetal medication can be administered through fetal intramuscular injection, umbilical vein injection, intra-amniotic injection, or fetal abdominal cavity injection. Lin et al*.* reported successful cases of repeated fetal abdominal and intra-amniotic injection of amiodarone for treating AFL (29). Munoz et al. reported a case of fetal intramuscular injection of digoxin for treating refractory SVT with fetal edema (30). However, considering the potential trauma to the fetus associated with direct medication, these approaches have not yet gained widespread acceptance and application in clinical practice.

### 3.3 Fetal transesophageal pacing

Stirnemann et al. reported a case of successful *in utero* transesophageal pacing for treatment of severe refractory tachyarrhythmias (31). The case was a fetus at 27 + 5 weeks’ gestation with AFL and fetal edema. After treatment with digoxin combined with flecainide and amiodarone, fetal edema worsened, accompanied by subcutaneous edema, persistent AFL, and tricuspid and mitral valve regurgitation. At 29 + 4 weeks’ gestation, fetal cardiac pacing via the esophagus was performed under fetoscopic guidance and ultrasound monitoring. Sinus rhythm was restored postoperatively, and there was no recurrence. In experienced cardiac centers, fetal transesophageal pacing can be considered as a salvage therapy for refractory tachyarrhythmias when medical treatment fails, but caution must be exercised regarding iatrogenic premature rupture of membranes and related complications, as well as the risk of ventricular fibrillation caused by transesophageal pacing.

## 4 Conclusion

Presently, the prenatal treatment of FTs is still mainly based on drug therapy. Transplacental transport drug therapy commonly employed as a first-line treatment. While drug therapy via placental transfer exposes the mother to the side effects and risks of antiarrhythmic medications, its efficacy may be limited, particularly in cases of fetal edema. The treatment of FTs depends on a comprehensive analysis and assessment of gestational age, evidence of fetal edema, and potential risks to the mother. Currently, there is no randomized clinical study demonstrating the superiority of one antiarrhythmic drug over another, but flecainide and sotalol (rather than digoxin) are increasingly becoming the first-line treatment for fetal SVT and AFL.

Another significant consideration is the current ethical challenges. As all prenatal treatment approaches involve passing through the maternal body due to the fetus being located within the uterus, they inevitably affect the mother to varying degrees, thus raising numerous ethical concerns. In 2017, the International Fetal Medicine and Surgery Society (IFMSS) together with the North American Fetal Therapy Network (NAFTNet) issued fundamental principles for fetal *in utero* therapy in response to emerging needs (32). These principles emphasize that the goal of fetal therapy is no longer solely focused on improving fetal survival rates but increasingly on the short- and long-term effects on maternal health. Therefore, before deciding on prenatal intervention for fetal arrhythmias, a comprehensive assessment should be conducted to weigh the benefits of restoring rhythm against the potential adverse effects of medications on both the mother and fetus, aiming to achieve the most favorable outcome, ensuring the safety of both mother and baby, and minimizing complications to the greatest extent possible.

Understanding the indications and clinical outcomes for prenatal treatment of FTs helps facilitate timely intervention and prevent adverse outcomes. However, current research on prenatal treatment of FTs mainly consists of retrospective clinical case studies, with only a few prospective, single-center studies, limited case numbers, and variations in medication methods, dosages, and efficacy among different research centers. Furthermore, systematic analysis of medication dosages and side effects are lacking. The FAST RCT trial, initiated in 2016, is a prospective randomized clinical trial investigating the treatment of fetal AFL and SVT. Its aim is to determine the effectiveness and safety of commonly used drug regimens administered via placental transfer in treating AFL and SVT in fetuses with or without edema (33). This trial aims to bridge the knowledge gap regarding drug efficacy and adverse effects on both pregnant patients and fetuses, ultimately establishing systematic treatment protocols for FTs. It is believed that once the results are published, they will provide valuable insights into individualized prenatal drug therapy for FTs.
